# Evidence that Enzyme Processivity Mediates Differential Aβ Production by PS1 and PS2

**DOI:** 10.2174/156720513804871480

**Published:** 2013-01

**Authors:** Sean A Pintchovski, Dale B Schenk, Guriqbal S Basi

**Affiliations:** Elan Pharmaceuticals, Inc., South San Francisco, CA 94080, USA

**Keywords:** Aβ, AICD, γ-secretase, NICD, PS1, PS2, processivity.

## Abstract

The γ-secretase complex cleaves the carboxy-terminal 99 residue (C99) fragment of the amyloid precursor protein (APP) to generate the amyloid-β (Aβ) peptide. The catalytic activity of this complex is mediated either by the presenilin-1 (PS1) or the presenilin-2 (PS2) subunit. *In vitro* and *in vivo* studies have demonstrated that PS1-containing complexes generate more total Aβ product than PS2-containing complexes, indicating greater cleavage activity by PS1-containing γ-secretase complexes at the APP γ-site. However, it remains untested whether γ-secretase cleavage at the APP ε-site, which precedes γ-site cleavage and produces the physiologically active APP intracellular domain (AICD), follows the same rule. Using a novel Swedish APP-GVP substrate to facilitate the parallel detection of Aβ and AICD products from PS1^-/-^/PS2^-/-^ cells co-transfected with either PS1 or PS2, we observed that while PS1 generates more total Aβ product than PS2, consistent with published reports, PS1 and PS2 unexpectedly generate equal amounts of AICD product. We also observed that PS1 and PS2 produce equivalent amounts of Notch intracellular domain (NICD), indicating equal cleavage activity at the Notch S3-site (the corollary of the APP ε-site). Our findings suggest that processivity differences between PS1 and PS2 underlie the differential production of Aβ peptide. Taken together these findings offer novel insights into γ-secretase biology and have important implications for therapeutically targeting γ-secretase

## INTRODUCTION

γ-Secretase is an integral membrane protease that mediates the intra-membrane cleavage of over 60 known substrates involved in a range of physiological processes [1-3]. Each multi-subunit γ-secretase complex is comprised of Nicastrin, Aph-1, Pen-2, with either PS1 or PS2 as the catalytic core [4-9]. Amyloid precursor protein (APP) is the most well studied substrate of γ-secretase owing to its central role in the generation of Aβ peptide, which is strongly implicated in the pathophysiology of AD. 

*In vitro* cellular and biochemical studies [8-12], as well as *in vivo* loss of function studies [13-18] have demonstrated that PS1-containing complexes generate significantly more Aβ peptide from the APP substrate than PS2-containing complexes. As a result, the majority of studies have focused on PS1 in their efforts to better understand γ-secretase biology and to identify ways to inhibit or modulate its activity for the treatment of AD. Relatively little is therefore known about the biology of PS2 and to what extent PS1- and PS2-containing complexes might differ in how they process substrates. 

The underlying basis for the observed difference between PS1- and PS2-mediated Aβ production remains unclear. PS1 and PS2 exhibit similar expression levels and a highly overlapping expression pattern throughout both the rat and human adult brain, as well as peripheral tissues [19-24]. One possible explanation is that the difference in Aβ production is due to a mechanistic difference in the way that PS1 and PS2 recognize and/or cleave APP substrate. A parsimonious explanation for the greater production of Aβ by PS1 is that PS1-containing γ-secretase is a more active enzyme than PS2-containing γ-secretase [11]. 

Recent work indicates that γ-secretase cleaves the transmembrane domain of APP in a processive fashion [25, 26], whereby the AICD is released first by γ-secretase cleavage at the APP ε-site and the predominant secreted product, Aβ_40_, is subsequently released as a result of processive cleavages. Furthermore, the final cleavage site in the transmembrane domain is limited by a charged basic residue at the luminal-transmembrane boundary of substrates [27], culminating in the cleavage of APP at the intramembrane γ-site corresponding to the carboxy-terminus of Aβ_40_. Since PS1 produces more Aβ than PS2, the above findings would suggest that PS1 also generates more AICD than PS2. However, with the exception of one report [10], little is known about the relative activity of PS1- and PS2-containing γ-secretase complexes in the initial APP substrate ε-site cleavage event leading to AICD release.

In this study we introduced an APP-GVP fusion substrate into a PS1^-/-^/PS2^-/-^ fibroblast line and, by co-transfecting either PS1 or PS2 to transiently reconstitute γ-secretase activity, provide direct evidence that PS1 and PS2 generate equal levels of AICD product. By extension, this finding indicates that PS1 and PS2 yield different AICD:Aβ product ratios since it is well documented (and we show in our system) that PS1 generates more total Aβ product than PS2. Finally, we observe that PS1 and PS2 also generate equal levels of NICD product, indicating that these two enzymes have similar cleavage activities at the S3-site of Notch substrate. This study therefore reveals several important insights into PS1- and PS2-dependent γ-secretase substrate processing and suggests new approaches for the development of therapeutically effective γ-secretase inhibitors and modulators.

## METHODS

### Plasmids

Full-length Swedish APP-GVP was subcloned using the APP C99-GVP backbone (previously reported [28]). Briefly, the ectodomain of Swedish APP695 was PCR amplified and blunt-end ligated in-frame into linearized APP C99-GVP using the In-Fusion PCR Cloning System (Clontech). Swedish APP695, NotchΔE, NotchΔEΔC, Human PS1, and Human PS2 cDNA constructs were previously described [29-31].

### Cell Culture and Transient Transfection

Mouse fibroblasts derived from PS1^-/-^/PS2^-/-^ embryos [14] were grown at 37°C under 7.5% CO_2_ in Dulbecco’s Modified Eagle Medium (DMEM) containing 10% fetal bovine serum (FBS) and 2 nM L-Glutamine (Gibco /Invitrogen). We used the Nucleofector II device (Amaxa) for all transfections. Briefly, PS1^-/-^/PS2^-/-^ cells were harvested by trypsin digest, separated into aliquots of ~2.5x10^6^ cells (one aliquot per condition), and pelleted by centrifugation at 90×g for 10 min. Cell pellets were then rinsed with 5 mL of warm RPMI Medium 1640 (Gibco/Invitrogen) and centrifuged again at 90×g for 5 min. Cell pellets were carefully resuspended in 100 μL Solution R and 4-5 μg of endotoxin-free DNA was added (including 1 μg of β-Galactosidase DNA to normalize transfection efficiency). This cell-DNA mixture was immediately electroporated with the preset T-020 program, and 1 mL of warm RPMI was then immediately added to the cells. 2-5 min after addition of RPMI the mixture was transferred into 5-10 mL of DMEM with 10% FBS, and seeded at ~50,000 per well on poly-D-Lysine-coated 96-well plates (BD Biosciences). After ~4 h the old media was aspirated and fresh media containing either DMSO, the γ-secretase inhibitor N-[N-(3,5-difluorophena cetyl)-L-alanyl]-S-phenylglycine t-butyl ester (DAPT, 5 μM) [32], or the γ-secretase inhibitor LY411575 (100 nM) [33] was added for overnight incubation.

### Luciferase Reporter Signaling Assays

NotchΔE substrate (2 μg) was co-transfected with CBF-*Luciferase* reporter gene (1 μg) [29, 34] and Swedish APP-GVP substrate (2 μg) was co-transfected with UAS-*Luciferase* reporter gene (1 μg) [28]. The *β-Galactosidase* expression plasmid pCMV-LacZ (1 μg, Life Technologies) was also co-transfected with each condition to normalize for transfection efficiency. Each condition was tested in quadruplicate, and measured values were averaged and normalized for total PS1 or PS2 expression levels by Western blot and densitometric quantification of mature Nicastrin levels (using a standard curve).

### Enzyme-Linked Immunosorbent Assays

Aβ1-x represents any Aβ peptide equal to or longer in length than Aβ1-23 including Aβ38, Aβ40, and Aβ42. Aβ1-x was quantified using antibody 266 (epitope Aβ16-23) for capture and biotinylated antibody 2H3 (epitope Aβ4-7) for detection, and both antibodies have been described previously [28]. NICD was quantified using antibody 9F3 (N-terminal neo-epitope, [31]) for capture and a biotinylated anti-HA antibody for detection. For AICD and NICD ELISAs, cell lysates were harvested in 1X Passive Lysis Buffer, plates were briefly centrifuged to pellet debris, and recovered lysate was added to each well of the appropriate ELISA plate. For Aβ1-x ELISAs, 90 μL of conditioned medium was recovered after overnight incubation of transfected cells (immediately before cell lysis in the case of AICD ELISAs). Each condition was tested in quadruplicate, and measured values were averaged and normalized for total PS1 or PS2 expression levels by Western blot and densitometric quantification of mature Nicastrin levels (using a standard curve). 

### Western Blot Detection

Tissue culture plates were washed with cold Tris-buffered saline (TBS) and homogenized in 1X Passive Lysis Buffer (0.1% Triton X-100, EDTA-free protease inhibitor mixture (Sigma) in TBS). All samples were solubilized at 4°C for 1 h and cleared by centrifugation at 14,000xg. Aliquots of the supernatant were boiled for 5 min in Laemmli sample buffer and resolved on 10–20% Tris-Tricine SDS-PAGE gels (Invitrogen). The gels were transferred with the iBlot system (Invitrogen), and membranes were blotted with appropriate antibodies and visualized with standard ECL substrate (Pierce). Antibodies used: AICD-GVP/APP (Sigma, A8717), Nicastrin (Abcam, AB3444), NICD/Notch (Upstate, 07220), anti-HA to detect NICDΔC-HA (Roche, 11867423001). Semi-quantitative densitometric measurements were performed using standard curves, run in parallel with the samples, generated by diluting lysates from HEK293 cells transfected with the corresponding substrate.

### Statistical Analyses

Student’s paired *t* tests and one-way ANOVA with *post hoc* Tukey’s *t* tests were performed with Prism (GraphPad Software).

## RESULTS

### PS1 and PS2 Comprised γ-Secretase Complexes Exhibit Similar Activity for the APP ε-Site

**Fig. (1):**
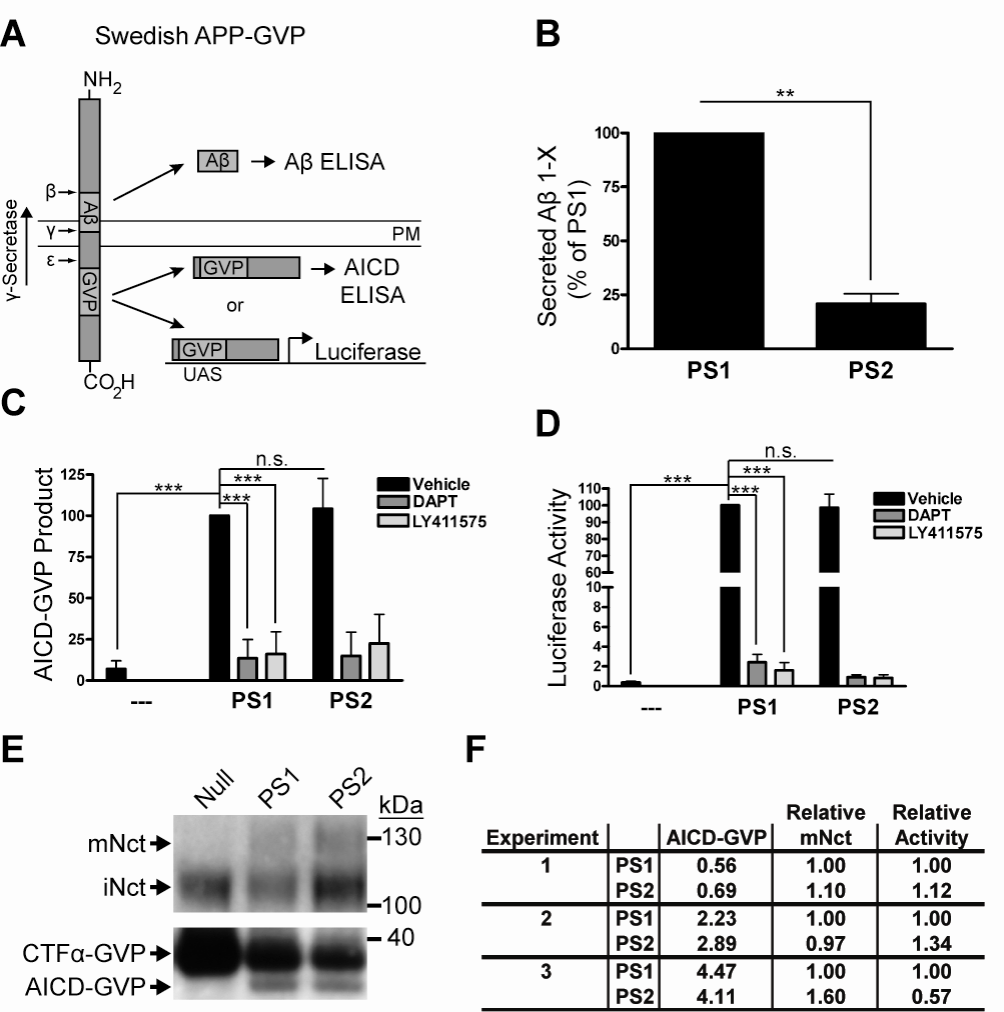
PS1 and PS2 generate equal amounts of AICD-GVP product. **A**) The newly created Swedish APP-GVP fusion protein substrate (927 amino acids) contains a GVP domain within the AICD. After the initial cleavage by the BACE enzyme at the Swedish APP-GVP β-site, the remaining c-terminal APP-GVP fragment in the plasma membrane (PM) is cleaved by γ-secretase in the indicated direction. The GVP domain is composed of a GAL4 DNA-binding domain and a VP16 transcriptional transactivation domain that, when translocated into the nucleus after γ-secretase cleavage at the APP ε-site, drives UAS-dependent *Luciferase* enzyme expression in a linear and quantifiable manner. The AICD-GVP product can also be detected by ELISA and Western blot. This permits the simultaneous detection of AICD-GVP and Aβ product levels from the same substrate. **B**) Aβ ELISAs performed on conditioned media from transfected PSdKO MEFS demonstrate that PS1 generates more Aβ than PS2, consistent with the literature, thereby validating the use of this substrate and this system. Both conditions were normalized for transfection efficiency, total Swedish APP-GVP substrate levels, and functional γ-secretase levels (Fig. **1E**, **1F**). PS2 values are represented as a percentage (mean ± SD) of the value for PS1. N=3, Student’s *t* test: **p<0.01. **C**) AICD-GVP ELISAs performed on cell lysates from transfected PSdKO MEFs demonstrate that PS1 and PS2 generate approximately equal amounts of AICD-GVP product (DMSO vehicle control), The structurally distinct γ-secretase inhibitors DAPT and LY411575 inhibit AICD-GVP production almost completely down to the background level observed with the empty vector control, indicating that these PS1- and PS2-mediated effects are γ-secretase activity dependent. All conditions were normalized for transfection efficiency, total Swedish APP-GVP substrate levels, and functional γ-secretase levels. All values are percentages (mean ± SD) of the value for PS1. N=3, one-way ANOVA and *post hoc* Tukey’s *t* tests: ***p<0.001; n.s., not statistically significant. **D**) Swedish APP-GVP substrate was co-transfected with a UAS-*Luciferase* reporter gene into PSdKO MEFS and AICD-GVP product levels were measured by *Luciferase* assays performed on the cell lysates. This demonstrated that PS1 and PS2 generate approximately equal amounts of AICD-GVP product (DMSO vehicle control). The structurally distinct γ-secretase inhibitors DAPT and LY411575 inhibit *Luciferase* activity almost completely down to the background level observed with the empty vector control, indicating that these PS1- and PS2-mediated effects are γ-secretase activity dependent. All conditions were normalized for transfection efficiency, total Swedish APP-GVP substrate levels, and functional γ-secretase levels. All values are percentages (mean ± SD) of the value for PS1. N=3, oneway ANOVA and *post hoc* Tukey’s *t* tests: ***p<0.001; n.s., not statistically significant. **E**) *Top panel*. Western blot detection of levels of mature Nicastrin, which is only present in fully competent γ-secretase complexes containing all subunits [35], in PS1- and PS2- expressing MEFs. Blot images are representative of N=3 experiments. This confirms the PS1^-/-^/PS2^-/-^ MEF genotype and demonstrates that AICD-GVP and Aβ production is rescued by PS1 or PS2. mNct, mature Nicastrin; iNct, immature Nicastrin. *Bottom panel*. Western blot (using antibody against APP C-terminus) demonstrates saturating levels of APP C-terminal fragment (CTF) substrate in each condition. The predominant CTF isoform generated from full-length APP substrate is CTFα, here producing CTFα-GVP. Blot images are representative of N=3 experiments. All samples were normalized during loading for both total protein and transfection efficiency. **F**) Densitometric quantification of mature Nicastrin levels and AICD-GVP product levels was performed (using standard curves, not shown). Relative mature Nicastrin levels were used to calculate the relative AICD-GVP generating activities of PS1 and PS2. As with the AICD-GVP ELISAs and *Luciferase* assays, these Western blots indicate that PS1 and PS2 produce relatively equal amounts of AICD-GVP product.

In order to simultaneously measure both AICD and Aβ product levels from the same substrate we first constructed a Swedish APP substrate containing a Gal4-VP16 (GVP) domain within the AICD region. The GVP domain serves to stabilize the normally labile AICD by an unknown mechanism, permitting the detection of AICD-GVP product levels by ELISA or *Luciferase* assay (Fig. **1A**). PS1^-/-^/PS2^-/-^ double knock-out mouse embryonic fibroblasts (PSdKO MEFs) were transfected with this Swedish APP-GVP substrate and either empty vector, wild-type human PS1, or wild-type human PS2 and each transfection was incubated overnight in media containing either DMSO vehicle control, or the γ-secretase inhibitors DAPT and LY411575. Conditioned media was then harvested for Aβ ELISA at the same time that cell lysates were harvested for AICD-GVP ELISA or *Luciferase* assay. Consistent with previous reports [10, 11, 13], the Aβ ELISA demonstrated that PS1 generates approximately five-fold more Aβ than PS2 (Fig. **1B**), thereby validating our substrate and cell-based system. Interestingly, and in contradiction with observations reported by Bentahir *et al.* [10], the same PS1- and PS2-expressing cells that generated different amounts of Aβ generated equal amounts of AICD-GVP as measured by both a neo-epitope ELISA specific for the N-terminus of AICD (Fig. **1C**) and a *Luciferase* reporter gene assay (Fig. **1D**). In addition, AICD-GVP levels were measured by Western blot and semi-quantitative densitometry to further confirm that PS1 and PS2 generate equal amounts of AICD-GVP product (Fig. **1E**, bottom panel; Fig. **1F**).

To verify that the AICD-GVP product levels measured here did not simply result from some artifact unique to the novel Swedish APP-GVP substrate, we also tested the previously described APP C99-GVP substrate [28]. Similar results were obtained with APP C99-GVP substrate (data not shown), indicating that different APP substrates yield the same result. All experiments were carefully normalized for transfection efficiency by including equal amounts of β-Galactosidase expression plasmid and assaying for β-Gal activity in the cell lysates. Expression levels of functional PS1- and PS2-containing γ-secretase complexes were normalized by Western blot and densitometric quantification of mature Nicastrin levels, a proxy for functional γ-secretase levels [35] (Fig. **1E**, top panel; Fig. **1F**). In addition, levels of the APP-GVP C-terminal fragment (CTF), the APP metabolite that serves as the immediate substrate for γ-secretase, were quantified by Western blot and densitometry to confirm saturating levels of substrate in all conditions (Fig. **1E**, bottom panel; Fig. **1F**; Supplemental Fig. **1**). Together these findings indicate that PS1 and PS2 generate different amounts of Aβ product yet equal amounts of AICD-GVP product. 

### PS1 and PS2 Exhibit Equal Specific Activity for the Notch S3-Site

**Fig. (2):**
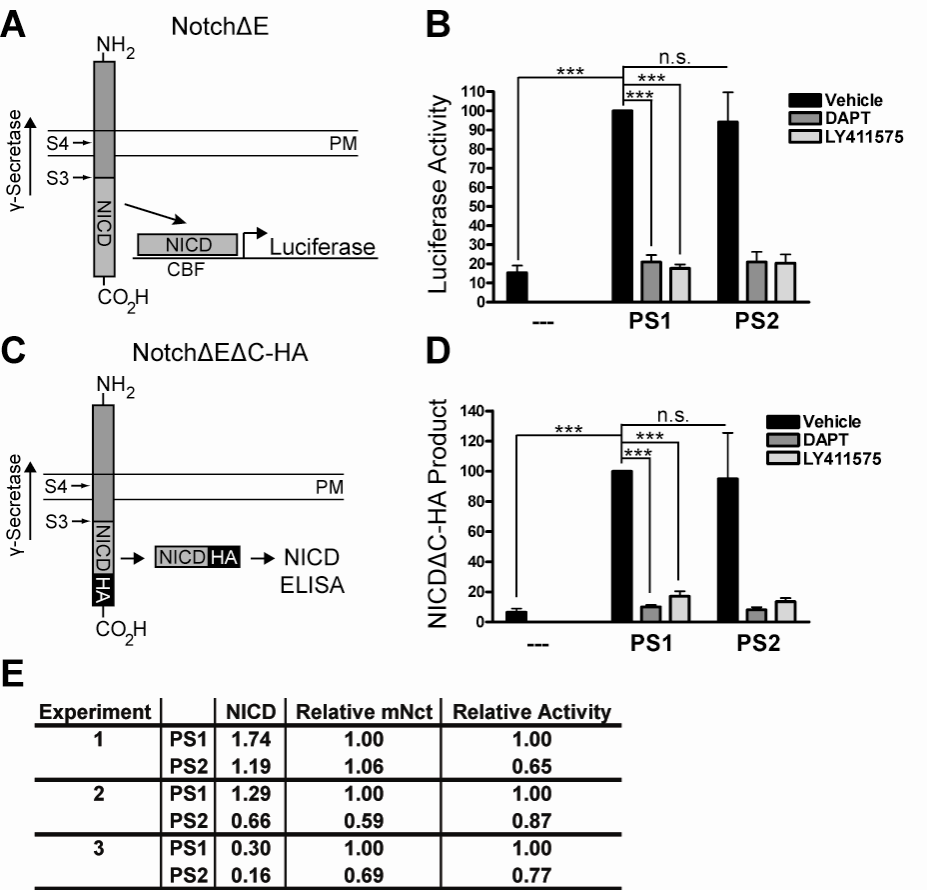
PS1 and PS2 generate equal amounts of NICD product. **A**) NotchΔE substrate was co-transfected with a CBF-*Luciferase* reporter gene into PSdKO MEFS and NICD product levels were measured by *Luciferase* assays performed on the cell lysates. The direction of γ- secretase cleavage activity along the NotchΔE substrate is indicated. PM, plasma membrane. **B**) *Luciferase* assays demonstrated that PS1 and PS2 generate approximately equal amounts of NICD product (DMSO vehicle control). The structurally distinct γ-secretase inhibitors DAPT and LY411575 inhibit *Luciferase* activity down to the background level observed with the empty vector control, indicating that these PS1- and PS2-mediated effects are γ-secretase activity dependent. All conditions were normalized for transfection efficiency, total NotchΔE substrate levels, and functional γ-secretase levels. All values are percentages (mean ± SD) of the value for PS1. N=3, one-way ANOVA and *post hoc* Tukey’s *t* tests: ***p<0.001; n.s., not statistically significant. **C**) NotchΔEΔC-HA substrate was used to detect NICDΔC-HA product levels by ELISA. The direction of γ-secretase cleavage activity along the NotchΔEΔC-HA substrate is indicated. PM, plasma membrane. **D**) NICDΔC-HA ELISAs performed on cell lysates from transfected PSdKO MEFs demonstrate that PS1 and PS2 generate approximately equal amounts of NICDΔC-HA product (DMSO vehicle control). The structurally distinct γ-secretase inhibitors DAPT and LY411575 inhibit NICDΔC-HA production almost completely down to the background level observed with the empty vector control, indicating that these PS1- and PS2-mediated effects are γ-secretase activity dependent. All conditions were normalized for transfection efficiency, total NotchΔEΔCHA substrate levels, and functional γ-secretase levels. All values are percentages (mean ± SD) of the value for PS1. N=3, one-way ANOVA and *post hoc* Tukey’s *t* tests: ***p<0.001; n.s., not statistically significant. **E**) Western blot and densitometric quantification of mature Nicastrin levels and NICD product levels was performed (using standard curves, not shown). Mature Nicastrin levels were used to calculate the relative NICD generating activities of PS1 and PS2. As with the NICD ELISAs and *Luciferase* assays, these Western blots indicate that PS1 and PS2 produce relatively equal amounts of NICD product.

Recent work suggests that γ-secretase cleaves Notch in a processive manner similar to that of APP [36]. To determine whether PS1 and PS2 display equal cleavage activities for the S3-site of Notch, as observed for the ε-site of APP, we tested different Notch substrates again using the PS1^-/-^PS2^-/-^ mouse embryonic fibroblast system and measured NICD product levels generated by PS1 and PS2. First, PSdKO MEF cells were transfected with NotchΔE substrate and either empty vector, wild-type human PS1, or wild-type human PS2. Each condition was incubated over-night in media containing DMSO vehicle control, DAPT, or LY411575. Cell lysates were then harvested to perform *Luciferase* assays as a read-out of NICD product levels (Fig. **2A**). This demonstrated that PS1 and PS2 generate equal amounts of NICD in a γ-secretase-dependent manner (Fig. **2B**). 

To verify this finding, we next used the amino- and carboxy-terminally truncated NotchΔEΔC-HA substrate [31], which incorporates a c-terminal HA-epitope tag to facilitate NICD detection by ELISA (Fig. **2C**). Again, this assay indicated that PS1 and PS2 generate equal amounts of NICD in a γ-secretase-dependent manner (Fig. **2D**), consistent with previous reports [10]. In addition, NICD levels were measured by Western blot and densitometry to further confirm that PS1 and PS2 generate approximately equal amounts of NICD product (Fig. **2E**). Importantly, and as before, all conditions were carefully normalized for transfection efficiency, total substrate levels, and total functional γ-secretase levels (Supplemental Fig. **2**). These results suggest that PS1 and PS2 generate equivalent amounts of NICD product from Notch substrate.

## DISCUSSION

In this study multiple methods were used to characterize the initial cleavage of APP and Notch, the two best characterized γ-secretase substrates, by PS1- and PS2-containing complexes. We present direct evidence that PS1 and PS2 generate equal amounts of AICD product (Fig. **1C**, **1D**, **1E**, **1F**) from an APP fusion protein substrate, as well as equal amounts of NICD product (Fig. **2B**, **2D**, **2E**) from two constitutive Notch substrates. Our results offer new insights into the mechanistic basis for the differential production of Aβ by PS1 and PS2 γ-secretases.

The equivalent activity of PS1- and PS2-comprised γ-secretase complexes for the APP substrate ε-site indicates that PS1 and PS2 yield different AICD:Aβ product ratios since we have shown in our system (Fig. **1B**) and it is well documented that PS1 generates more Aβ than PS2 [8-18]. Previous work indicates that γ-secretase cleaves APP in a processive manner such that ε-site cleavage (resulting in AICD release) precedes γ-site cleavage (resulting in Aβ release) [25, 37]. In addition, several distinct PS1 and PS2 familial mutations influence the relative production of AICD and Aβ [10, 38, 39], supporting the notion that the production of AICD and Aβ are dissociable. Taken together, these findings suggest that processivity differences between PS1 and PS2 could account for their differential Aβ production, consistent with the finding with PS1 FAD mutations [39]. 

There are several possible explanations for the mechanistic bases underlying differential AICD:Aβ production. First, Lee *et al.* [40] identified the variable contributions of different Aph1 isoforms to PS1- and PS2-mediated Aβ production in Sf9 cells. Specifically, they observed equal Aβ production by PS1 and PS2 in the presence of Aph1a, while Aph1b lowered Aβ production only from PS1 complexes by ~60% relative to Aph1a. We did not investigate the role of endogenous Aph1 isoforms in our mammalian PSdKO MEF cells. Another possibility is that some specific (perhaps yet unidentified) auxiliary γ-secretase subunit, which regulates γ-secretase cleavage properties, might be selectively recruited to either PS1- or PS2-containing complexes. Indeed, the auxiliary subunit TMP21 modulates γ-site but not ε-site cleavage of APP, Notch, and Cadherin [41]. However, as with most reports, the study of TMP21 only examined PS1-containing complexes so it remains unclear whether TMP21 or some other factor might be important for regulating the different PS1- and PS2-mediated AICD:Aβ product ratios. In addition, factors such as enzyme and substrate sub-cellular localization as well as pH have been reported to influence microheterogeneity in ε-site cleavage specificity [42], although no influence on the AICD:Aβ (i.e., ε-site vs. γ-site) product ratio has been noted. 

One potential caveat from our study is that we did not test whether the GVP domain alters the structure or the position of the Swedish APP-GVP substrate within the plasma membrane. Such changes to the substrate may result in non-physiological γ-secretase activity and AICD generation. However, since we also provide evidence that PS1 and PS2 generate equal amounts of NICD product from the non-modified, physiological NotchΔE substrate (discussed below) it is unlikely that the GVP domain confounded the interpretation of our data. 

It is also worth noting that the choice of system used in studies investigating γ-secretase activity can contribute to differences or discrepancies between reports from different groups. For example, our findings regarding PS1- and PS2-mediated NICD production are in agreement with those of Bentahir *et al.* [10], consistent with the fact that both observations are from cell-based assays. However, in contrast to our finding regarding equivalent PS1- and PS2-mediated AICD production in transiently transfected cells, using cell-free extracts Bentahir *et al.* observed AICD production only by PS1 γ-secretase complexes. Furthermore, our findings regarding the different Aβ:AICD ratios generated by PS1- and PS2-containing γ-secretases contradict the report by Kakuda *et al.* [43]. Again, this may be due to an inherent difference between our cellular assay and their *in vitro* biochemical system. It is also possible that the different methodologies for normalizing PS1 and PS2 expression levels, and thus calculating their relative enzymatic activities, could contribute to this discrepancy. 

This study also used multiple techniques to provide direct evidence that PS1 and PS2 also generate equal amounts of NICD product (Fig. **2B**, **2D**, **2E**). In combination with our findings above, these observations are significant for the following reasons. First, they indicate that PS1 and PS2 have similar cleavage activities at the S3/ε-sites of at least two unique substrates, perhaps suggesting a common mechanism by which γ-secretase processes all of its substrates. Furthermore, Notch is cleaved by γ-secretase in a processive manner similar to that of APP [36], so it will be important for future work to determine whether PS1 generates more Notch P3 (also referred to as Notch-β [44, 45]) product than PS2 by successive cleavages culminating at the Notch S4-site, (the corollary to the APP γ-site), and whether Aph1 isoform heterogeneity contributes to processivity on Notch substrate. 

Second, given the especially critical role that NICD-mediated signaling plays in a range of physiological processes [46], our observation that PS1 and PS2 γ-secretases generate equivalent amounts of NICD and AICD product is of direct relevance for reducing the toxicity associated with γ-secretase targeted therapeutics. Consequently, we suggest that γ-secretase inhibitors that preferentially inhibit PS1-containing γ-secretase complexes (e.g., sulfonamides [30, 40]) represent a promising avenue for reducing Aβ production while leaving the generation of the physiologically critical AICD and NICD products relatively unaffected. 

In summary, our report reveals several important insights into PS1- and PS2-dependent γ-secretase substrate processing, with implications for the development of therapeutically effective γ-secretase inhibitors and modulators.

## SUPPLEMENTARY MATERIALS

Supplementary material is available on the publishers web site along with the published article.
