# Supplementary material for: Evidence that Enzyme Processivity Mediates Differential Aβ Production by PS1 and PS2
Source: Curr Alzheimer Res. 2013 Jan;10(1):4–10. doi: 10.2174/156720513804871480 (PMC3617584; doi:10.2174/156720513804871480)
Supplement: Supplementary file 1 [file CAR-10-4_SD1.pdf]

SUPPLEMENTARY MATERIAL

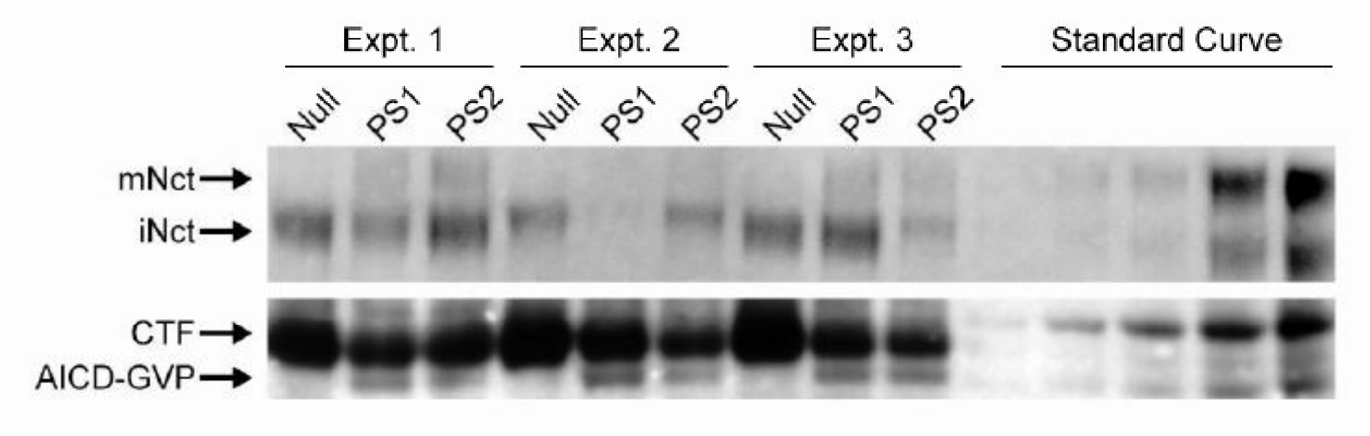

Supplementary Figure 1. Densitometric normalization data for AICD-GVP.

Top panel) Western blot detection of mature Nicastrin, which is only present in fully competent γ-secretase complexes [35], in PS1- or PS2-expressing PSdKO MEFs. A dilution curve of Nicastrin is included in the blot to facilitate semi-quantitative densitometry. mNct, mature Nicastrin ; iNct, immature Nicastrin.

Bottom panel) Western blot (using antibody against the APP C-terminus) demonstrates saturating levels of APP C-terminal fragment (CTF) substrate in each condition. A dilution curve containing CTF and AICD-GVP is included in the blot to facilitate semi-quantitative densitometry. All AICD-GVP values were normalized by total protein levels, transfection efficiency, and mNct levels to calculate relative activity of PS1 and PS2.

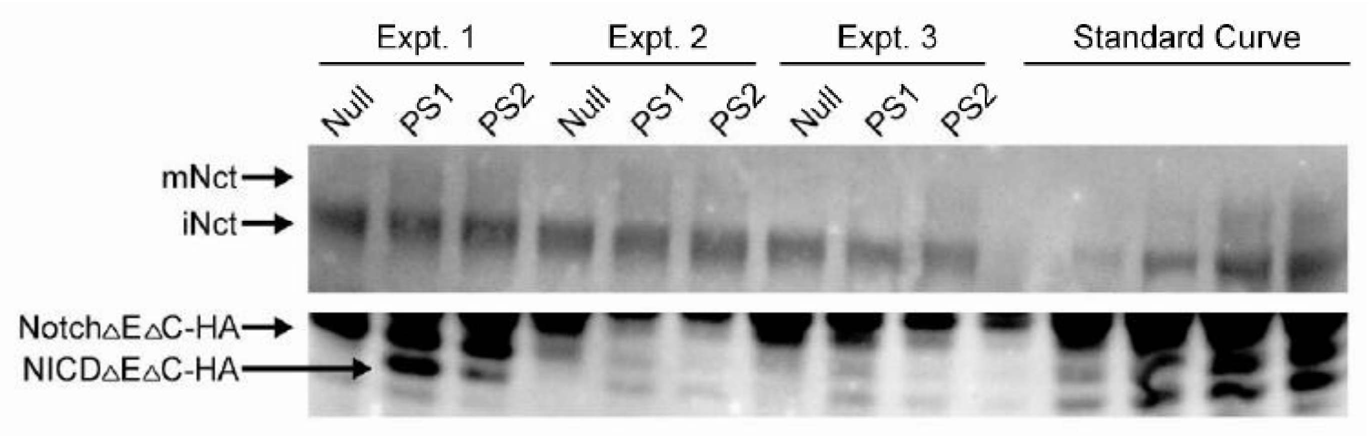

Supplementary Figure 2. Densitometric normalization data for NotchΔEΔC-HA.

Top panel) Western blot detection of mature Nicastrin in PS1- or PS2-expressing PSdKO MEFs. A dilution curve of Nicastrin is included in the blot to facilitate semi-quantitative densitometry.

Bottom panel) Western blot (using anti-HA antibody) demonstrates saturating levels of NotchΔEΔC-HA substrate in each condition. All NICDΔEΔC-HA values were normalized as for AICD-GVP in Supplementary Figure 1 (bottom panel).
